# Warfarin Pharmacogenetics: Challenges and Opportunities for Clinical Translation

**DOI:** 10.3389/fphar.2012.00183

**Published:** 2012-10-17

**Authors:** Nita A. Limdi

**Affiliations:** ^1^Department of Neurology, University of Alabama at BirminghamBirmingham, AL, USA; ^2^Department of Epidemiology, University of Alabama at BirminghamBirmingham, AL, USA

Despite its wide use over six decades, warfarin therapy remains challenging due its narrow therapeutic index. The multitude of factors interacting with warfarin makes it difficult to maintain anticoagulation within the target International Normalized Ratio (INR) range (Ageno et al., 2012). Even within this range the dose requirements vary as much as 20-fold between patients.

Deviations in INR control with frequent over and under-anticoagulation are common (Chiquette et al., 1998; Chamberlain et al., 2001; Ansell et al., 2007), are associated with poor outcomes with under-anticoagulation (increasing the risk of thrombosis) and over-anticoagulation (increasing the risk of serious or fatal hemorrhage), demanding that anticoagulation control be tightly regulated (Hylek and Singer, 1994; Hylek et al., 1996, 2000; Hylek, 2003; Wittkowsky, 2004; Wittkowsky and Devine, 2004; Hylek and Rose, 2009). These adverse outcomes have relegated warfarin to the “top 10 drugs” for adverse drug-related hospitalizations in the US (Budnitz et al., 2007, 2011). Between 2007 and 2009 warfarin accounted for 33% of drug-related hospitalizations for adverse events in the US (Budnitz et al., 2011). The risk for hemorrhage is particularly elevated when the INR exceeds four, as well as during the initial months of therapy. Therefore it is critical to achieve a safe and effective level of anticoagulation for patients starting warfarin.

Current guidelines for initiation of therapy provided by the American College of Chest Physicians (ACCP) allow flexibility in selecting a starting dose of warfarin, suggesting 5–10 mg. Although the ACCP guidelines recommend lower (2.5–5 mg) doses recognizing the influence of age, comorbidities, nutritional status, and drug interactions, these recommendations do not tailor dosing to individual patients (Ageno et al., 2012).

## Candidate Gene Studies

The recognition of genetic regulation of warfarin response has stimulated efforts aimed at quantifying this influence. The bulk of the evidence supports the influence of single nucleotide polymorphisms (SNPS) in two genes; Cytochrome P450 2C9 (*CYP2C9*; codes for the main enzyme involved in warfarin metabolism) and Vitamin K epoxide reductase complex1 (*VKORC1*; encodes the vitamin K–epoxide reductase protein, the target enzyme of warfarin). The influence of SNPs in *CYP2C9* and *VKORC1* on warfarin dose has been extensively assessed and reviewed (Wadelius et al., 2007, 2009; Limdi and Veenstra, 2008; Cavallari and Limdi, 2009; Klein et al., 2009). This evidence provided the basis for the recent warfarin package insert update by the United States Food and Drug Administration (FDA).

Moreover clinical algorithms that can enable dose prediction incorporating patient-specific genetic and clinical information have been developed and are freely available. Gage et al. (2008) have developed a dosing algorithm based on clinical and demographic factors (body surface area, age, target INR, amiodarone use, smoker status, race, current thrombosis) along with *CYP2C9* (*2, *3, *5, and *6), *VKORC1* (-1639/3673G>A), *GGCX* (rs11676382), and *CYP4F2* (V433M) polymorphisms. The algorithm is freely available at www.warfarindosing.org and allows calculation of warfarin dose based on clinical and demographic factors alone (if genotype is not available). Incorporation of novel and potentially important genetic variants (such as CYP2C9*8) can further improve dosing prediction in African American patients (Cavallari et al., 2010; Cavallari and Perera, 2012).

As demonstrated by multiple studies, including the work of the International Warfarin pharmacogenetics Consortium (IWPC), dosing based on clinical/demographic factors alone improves prediction of stable therapeutic dose of warfarin (compared to the one-size-fits-all 5 mg/day dose), specifically in patients that need =7 mg/day or =3 mg/day. Furthermore inclusion of *CYP2C9* and *VKORC1* provide a substantial gain in improvement of dose prediction in 46% of patients (Klein et al., 2009). The www.warfarindosing.org also allows the user to compute the estimated dose requirements based on the IWPC algorithm.

Both pharmacogenetic algorithms (Gage et al., 2008; Klein et al., 2009) require detailed mathematical calculations to predict warfarin dose. A simpler alternative is to refer to the genotype-stratified warfarin dose table recently added to the warfarin label by the U.S. FDA. Although pharmacogenetic algorithms are most accurate, the genotype-stratified warfarin dose table provides a more accurate dose prediction than empiric dosing (Finkelman et al., 2011).

The Clinical Pharmacogenetics Implementation Consortium (CPIC) of the National Institutes of Health Pharmacogenomics Research Network has developed guidelines to assist clinicians in the interpretation and use of *CYP2C9* and *VKORC1* genotype data for estimating therapeutic warfarin dose to achieve an INR of 2–3, should genotype results be available to the clinician. These guidelines are published (Johnson et al., 2011) and periodically updated based on new developments in the field (http://www.pharmgkb.org/page/cpic).

## Genome-Wide Association Studies

To identify other genes/SNPs that can explain variability in warfarin dose two genome-wide association studies (GWAS) have been conducted. Among patients of European descent these studies have confirmed the influence of *VKORC1*, *CYP2C9*, and identified *CYP4F2* as the main genes associated with dose (Cooper et al., 2008; Takeuchi et al., 2009; Cha et al., 2010). The genome-wide significance of the latter association remains to be confirmed. These studies suggest that identification of common variants in other genes exhibiting influence of magnitude similar to that of *CYP2C9* and *VKORC1* is unlikely, at least in Whites.

As these known variants in candidate genes account for a smaller percent of the variability in warfarin dose among Blacks, the IWPC is leading a GWA analysis in Blacks. Preliminary results of the ongoing GWAs meta-analysis were presented at the 2011 American Heart Association meeting (Perera et al., 2011). A parallel effort is planned to conduct a GWAs meta-analysis for multiple population groups (Whites, Asians, African American, Japanese, and Middle-Eastern).

Despite these efforts a large portion (40% among White and 60% among Blacks) of the variability in warfarin dose remains unexplained. Perhaps emerging genotyping technologies such as exome sequencing or whole genome sequencing will reveal important clues that can explain the missing heritability. In addition to interrogating genetic variation ongoing and future studies must assess in detail environmental (diet, smoking alcohol, etc.) and lifestyle (compliance, exercise, etc.) factors with similar rigor. It is very likely that this along with gene-environment interactions holds the key to explaining the majority of the variability in response.

One group that remains unrepresented is the Hispanic/Spanish population. This is of great importance in the US as people of Hispanic descent accounted for more than half the growth in the population between 2000 and 2010 and account for 16.3% of the US population (surpassing Blacks who account for 12.3%; U.S. Census Bureau, 2010).

## Pressing Challenges

1. **Will pharmacogenetic/genomic interventions have an impact on clinically meaningful outcomes?**
  Although extensive research efforts have identified several genetic markers strongly associated with outcomes of interest and hailed them as promising tools, these proclamations are based mainly on associations rather than their evaluation as predictors. Moreover such evaluations must be based on clinically relevant hard-endpoints such as anticoagulation control, hemorrhage and health-care utilization and costs (Limdi and Veenstra, 2010).
  At the crux of this debate are three questions:
  a) Can a genetic risk factor (genetic marker) associated with an adverse (or beneficial) outcome be a clinically useful predictor of that outcome? (clinical validity)
    - *CYP2C9* and *VKORC1* genotypes are clinically useful predictors of warfarin dose in clinical trials (Anderson et al., 2007, 2012).
  b) Can incorporation of the genetic factor predict risk of the outcome more accurately than existing clinical models? (Clinical utility)
    - Incorporation of *CYP2C9* and *VKORC1* genotypes provided superior warfarin dose prediction compared with the clinical algorithm (or the fixed 5-mg dose algorithm; Klein et al., 2009; Anderson et al., 2007, 2012).
    - The updated FDA package insert provides easy to use genotype-stratified warfarin dose table. Although pharmacogenetic algorithms are most accurate, the genotype-stratified warfarin dose tables provide a more accurate dose prediction than empiric dosing (Finkelman et al., 2011).
  c) Will the outcome predicted for individuals be sufficiently different to warrant a change in treatment decisions? (degree of clinical utility)
    1) Incorporation of *CYP2C9* and *VKORC1* genotypes *improves prediction of warfarin doses* as confirmed by randomized clinical trials proving excellent insight into the effectiveness of utilizing pharmacogenetics in a real-world setting (Anderson et al., 2012).
    2) Whether the benefits of pharmacogenetic guidance of warfarin dosing would translate into *improved anticoagulation control* is being evaluated.
      - Anderson et al. showed that pharmacogenetic-dosing resulted in a higher percent time in target range (PTTR; 69 and 71% at 1 and 3 months compared to the control group (58 and 59% at 1 and 3 months; Anderson et al., 2012).
      - The improvement in PTTR achieved by pharmacogenetic-dosing (vs. standard dosing) is greater than that achieved by specialty anticoagulation clinics (vs. usual medical care). Importantly, these benefits in the pharmacogenetic cohort accrued in a setting where warfarin-treated patients were typically managed by standard protocol by an anticoagulation service/clinic.
    3) Observational cohort studies have demonstrated that possession of CYP2C9 variant allele increases the risk of hemorrhage (Aithal et al., 1999; Margaglione et al., 2000; Higashi et al., 2002; Limdi et al., 2008). Whether the increased risk can be mitigated and whether the benefits of pharmacogenetic guidance of warfarin dosing would translate into a decrease in risk of hemorrhage remains to be determined.
  Not unlike randomized clinical trials conducted to prove drug efficacy and attain FDA approval, pharmacogenetic tests/interventions are being held to higher standards. To gain acceptance (in practice with reimbursement) the test/intervention must demonstrate improvement in intermediate endpoints (e.g., PTTR for warfarin) and preferably hard-endpoints (e.g., hemorrhage risk reduction) in randomized clinical trials.
2. **When will this evidence be available? Are we there yet?**
  A number of clinical trials studies have assessed whether dose prediction and anticoagulation control are superior in patients receiving pharmacogenetically guided dosing vs. standard dosing or on clinical factors excluding genotype. However trials to date have been limited by study design issues (not blinded, use of historic control group) and limited sample size. Nonetheless these studies have provided valuable information on effect sizes and genotype-specific expectations which was valuable in the design of large ongoing randomized trials.
  1. The *Clarification of Optimal Anticoagulation through Genetics* (COAG; NCT00839657) trial is a multicenter double-blind, randomized trial aims to determine whether the use of genetic and clinical information for selecting the dose of *warfarin* in 1022 patients during the initial dosing period will lead to improvement in stability of anticoagulation at 4 weeks relative to a strategy that incorporates only clinical information (without genetics).
  2. The *EUropean Pharmacogenetics of AntiCoagulant Therapy* (EU-PACT; NCT01119300) trials are single-blinded randomized controlled trials aiming to assess the safety and clinical utility of genotype-guided dosing of the three main coumarins used in Europe: acenocoumarol, phenprocoumon, and warfarin. The warfarin arm will recruit 900 (UK and Sweden) warfarin patients to determine if genotype-guided dosing vs. standard dosing improves PTTR at 3 months.
  3. The *Genetic InFormatics Trial* (GIFT; NCT01006733) of Prevent DVT is a randomized controlled trial aiming to determine the benefit of genotype-guided dosing vs. clinically guided dosing among 1600 participants (age 65 years or older) in reducing the composite endpoint: non-fatal VTE, non-fatal major hemorrhage, death, and an INR > 4.0 at 1 month.
  4. *Pharmacogenetic-dosing of warfarin: a controlled randomized trial* is led by the Taiwan Warfarin Consortium which aims to determine whether genotype-guided dosing can improve safety (as measured by time to target INR and PTTR) of warfarin treatment in 600 participants compared to clinically guided dosing.
  5. *Warfarin Adverse Events Reduction for Adults Receiving Genetic Testing at Therapy Initiation* (WARFARIN; NCT01305148) is a randomized blinded interventional trial where 4300 patients (age > 65 years) are to be randomized to warfarin dosing based on the GenoSTAT test plus clinical factors, or clinical factors alone, using the warfarindosing.org website. The primary aim is to determine if genotype-guided therapy reduces the incidence of warfarin-related clinical events, including major hemorrhage and thromboembolic events at 30 days and in fewer hospitalizations and/or deaths compared to clinically guided therapy at 90 days compared to clinically guided therapy.
  Additional information on the trials below and others can be found at www.clinicaltrials.gov. These trials will provide a robust data for efficacy/effectiveness and cost effectiveness analysis and will provide the foundation for policy development.
  Although detractors claim pharmacogenetics/genomics in general has not yielded information to justify the investment of effort and funds, progress in the genomics/genetics arena has been maintained a rapid pace compared to other fields in medicine. For warfarin, the first report identifying the *CYP2C9* *2 polymorphism was published in 1994. With the discovery of *VKORC1* in 2004 the field burgeoned with investigations in multiple populations across the world documenting the effect of *CYP2C9* and *VKORC1* on warfarin dose, anticoagulation control, and risk of hemorrhage. Twenty years from the report identifying the *CYP2C9* *2 (10 years following the identification of *VKORC1*), the results of the first double-blinded randomized clinical trial (COAG trial) testing genotype-guided dosing intervention are expected to be available.
3. **Although the uptake of newly available oral anticoagulants is slow, a frequently raised question is “Will warfarin or warfarin pharmacogenetics matter in the coming years?”**
  The introduction of Dabigatran (DBG; October 2010), Rivaroxaban (2011), and Apixaban (awaiting approval) is changing the landscape of anticoagulation therapy (Melnikova, 2009; Weitz et al., 2012). Although the use of DBG is increasing (0.6 million users in the US; Johnson, 2012; Pradaxa, 2012), warfarin remains the most widely used oral anticoagulant (25 million users) (Schirmer et al., 2010; Altman and Vidal, 2011; Cabral et al., 2011; Wittkowsky, 2011; Tzeis and Andrikopoulos, 2012). The Practice INNovation And CLinical Excellence (PINNACLE) registry focusing on patients with atrial fibrillation (AF) reports among patients who received oral anticoagulation, 87.4% were treated with *warfarin* while just 12.6% were prescribed one of the two new oral anticoagulants (Cardiology, 2012).
  The oral anticoagulant market is expected to exceed $ 9 billion by 2014 (Melnikova, 2009), driven by demographics of the aging population and increased incidence of cardiovascular disease, and the uptake of newly approved agents. Although the market share of DBG (and the newer agents) is expected to increase, its uptake is hindered by lack of monitoring, reversibility, and expense. Almost 2 years after approval of DBG, warfarin remains the most widely used oral anticoagulant.

Despite the approval of four *CYP2C9*/*VKORC1* rapid throughput genotyping platforms by the U.S. FDA over the last decade clinical implementation of genotype-guided dosing is lagging. Although genotype-guided therapy improves dose prediction is recognized, evidence that such an intervention will improve anticoagulation control, reduce risk of adverse events and health-care costs is limited. Results of ongoing clinical trials are expected to address these issues and will perhaps provide the much needed impetus to reevaluate reimbursement for genetic testing and for wider implementation.

The challenges unique to pharmacogenomic efforts have created an intangible benefit for science and humanity. Of note, most genetic/genomic investigations have identified genes with small effect sizes. To enable these discoveries investigators stepping outside the conventional paradigm of lab-based investigative efforts formed consortia to build collaborations across laboratories, departments, institutions, countries, and continents. Investigators within these consortia sharing a common goal, pooled unpublished data, working with complete strangers, while maintaining enviable focus and relentless effort to advance science. Among the consortia, the IWPC has successfully brought together >100 investigators from >25 institutions across >10 countries providing valuable contributions (Klein et al., 2009; Limdi et al., 2010; Perera et al., 2011) to the pharmacogenetic literature and much needed insight to inform the design of ongoing clinical trials in five short years.

One has to but conduct a PubMed search for GWAS for their favorite disease/phenotype and scroll through the authors and contributors list to understand the magnitude of such efforts, the network of collaborators created and progress made. These collaborations will continue (beyond the single publication) to advance science and enable discoveries beyond what can be gaged solely by investments; past, current, and future.
